# Utomilumab in Patients With Immune Checkpoint Inhibitor-Refractory Melanoma and Non-Small-Cell Lung Cancer

**DOI:** 10.3389/fimmu.2022.897991

**Published:** 2022-08-02

**Authors:** David S. Hong, Ajay K. Gopal, Alexander N. Shoushtari, Sandip P. Patel, Aiwu R. He, Toshihiko Doi, Suresh S. Ramalingam, Amita Patnaik, Shahneen Sandhu, Ying Chen, Craig B. Davis, Timothy S. Fisher, Bo Huang, Kolette D. Fly, Antoni Ribas

**Affiliations:** ^1^ Department of Investigational Cancer Therapeutics, Division of Cancer Medicine, University of Texas MD Anderson Cancer Center, Houston, TX, United States; ^2^ National Cancer Center Hospital East, Kashiwa, Seattle, WA, United States; ^3^ Melanoma Service, Department of Medicine, Memorial Sloan Kettering Cancer Center, New York, NY, United States; ^4^ University of California San Diego Moores Cancer Center, La Jolla, CA, United States; ^5^ Lombardi Comprehensive Cancer Center, Georgetown University Medical Center, Washington, DC, United States; ^6^ National Cancer Center Hospital East, Kashiwa, Chiba, Japan; ^7^ Department of Hematology and Medical Oncology, Winship Cancer Institute, Emory University, Atlanta, GA, United States; ^8^ START San Antonio, San Antonio, TX, United States; ^9^ Department of Medical Oncology, Peter MacCallum Cancer Centre and the University of Melbourne, Melbourne, VIC, Australia; ^10^ Pfizer Oncology, San Diego, CA, United States; ^11^ Pfizer Oncology, Groton, CT, United States; ^12^ Department of Medicine, Jonsson Comprehensive Cancer Center, University of California Los Angeles, Los Angeles, CA, United States

**Keywords:** utomilumab, 4-1BB/CD137, immune checkpoint inhibitor, melanoma, NSCLC

## Abstract

**Section Head:**

Clinical/translational cancer immunotherapy

**Background:**

The goal of this study was to estimate the objective response rate for utomilumab in adults with immune checkpoint inhibitor (ICI)-refractory melanoma and non–small-cell lung cancer (NSCLC).

**Methods:**

Utomilumab was dosed intravenously every 4 weeks (Q4W) and adverse events (AEs) monitored. Tumor responses by RECIST1.1 were assessed by baseline and on-treatment scans. Tumor biopsies were collected for detection of programmed cell death ligand 1, CD8, 4-1BB, perforin, and granzyme B, and gene expression analyzed by next-generation sequencing. CD8+ T cells from healthy donors were stimulated with anti-CD3 ± utomilumab and compared with control.

**Results:**

Patients with melanoma (n=43) and NSCLC (n=20) received utomilumab 0.24 mg/kg (n=36), 1.2 mg/kg (n=26), or 10 mg/kg (n=1). Treatment-emergent AEs (TEAEs) occurred in 55 (87.3%) patients and serious TEAEs in 18 (28.6%). Five (7.9%) patients discontinued owing to TEAEs. Thirty-two (50.8%) patients experienced treatment-related AEs, mostly grade 1–2. Objective response rate: 2.3% in patients with melanoma; no confirmed responses for patients with NSCLC. Ten patients each with melanoma (23.3%) or NSCLC (50%) had stable disease; respective median (95% confidence interval, CI) progression-free survival was 1.8 (1.7–1.9) and 3.6 (1.6–6.5) months. Utomilumab exposure increased with dose. The incidences of antidrug and neutralizing antibodies were 46.3% and 19.4%, respectively. Efficacy was associated with immune-active tumor microenvironments, and pharmacodynamic activity appeared to be blunted at higher doses.

**Conclusions:**

Utomilumab was well tolerated, but antitumor activity was low in patients who previously progressed on ICIs. The potential of 4-1BB agonists requires additional study to optimize efficacy while maintaining the tolerable safety profile.

## Highlights

### What Is Already Known on This Topic

The ability of 4-1BB/CD137 agonist monoclonal antibodies (mAb) to enhance T-cell mediated control of tumor growth has been known for almost 25 years, yet clinical application of this mechanism is hampered by the uncertain relationship between agonist dose, potency, and safety.

### What This Study Adds

We report clinical and translational results from patients with non-small-cell lung cancer (NSCLC) or melanoma dosed once every 4 weeks with either 0.24 mg/kg or 1.2 mg/kg of single-agent utomilumab, a 4-1BB/CD137 agonist mAb. The dose levels tested were well-tolerated but clinical efficacy was low. Analysis of gene expression in tumor biopsies suggested utomilumab efficacy was associated with immune-active tumor microenvironments, and pharmacodynamic activity may be reduced at higher doses. *In vitro* models of stimulated human T cells showed loss of pharmacodynamic activity at higher doses of utomilumab.

### How This Study Might Affect Research, Practice or Policy

Future clinical development strategies for 4-1BB agonist mAbs should explore approaches to optimize efficacy while maintaining the tolerable safety profile.

## Background

Cancer immunotherapy, notably monoclonal antibody (mAb) antagonists of the programmed cell death protein 1 (PD-1)/programmed cell death-ligand 1 (PD-L1) pathway, has shown significant promise for the treatment of a variety of solid tumor types, including non–small-cell lung cancer (NSCLC) (1) and melanoma (2). Only a small percentage of patients respond to PD-1/PD-L1 therapy, however, and those who do not respond represent a significant unmet need. In this respect, use of co-stimulatory proteins to activate and enhance anticancer immune responses has generated substantial interest (3). 4-1BB/CD137 is a co-stimulatory receptor of the TNF receptor superfamily induced upon activation in T cells, natural killer (NK) cells, and dendritic cells (4–8). It mediates immune cell proliferation and survival, cytokine production (4, 5, 9, 10), formation of immunologic memory, and sustained T-cell immune responses (11–13), as well as increased antibody-dependent cell-mediated cytotoxicity in Fc-receptor–activated NK cells (14). *In vivo* engagement of 4-1BB/CD137 by agonistic antibodies was shown to enhance antitumor immune responses and tumor regression in preclinical tumor models (4, 5, 15, 16).

The hypothesis that such immune enhancement would be clinically beneficial has led to evaluation of 4-1BB/CD137 agonists across multiple cancer types (16, 17). Urelumab, a fully human, non-ligand binding, CD137 agonist IgG4 monoclonal antibody (mAb), was the first to show promise in solid tumors in a phase I/II trial (18). A phase II trial in metastatic melanoma was terminated in 2009 due to hepatotoxicity (19), however, and identification of a safe and effective application for urelumab remains an area of active investigation (20) (NCT03792724, NCT02845323, NCT02451982). Utomilumab (PF-05082566), the second 4-1BB agonist mAb to enter clinical development, is a fully human immunoglobulin (Ig) G2 agonist monoclonal antibody that was well tolerated by patients with advanced solid tumors (NCT01307267) (21, 22). Additional studies of utomilumab in combination with rituximab (targets CD20, 23), pembrolizumab (inhibits PD-1, 24) or mogamulizumab (inhibits CCR4, 25) confirmed the safety and tolerability of utomilumab but did not identify a setting in which it could produce differentiated clinical benefit. Recent structural analyses of 4-1BB/CD137 bound to the endogenous 4-1BBL trimer indicated how utomilumab, but not urelumab, blocked 4-1BB interactions with 4-1BBL (23, 24). Cell based assays suggested that the interaction with utomilumab with 4-1BB was associated with milder agonist activity compared to urelumab (23). The identification of safe and effective 4-1BB agonists remains an active area of research, with over 25 clinical studies listed on clinicaltrials.gov as of 4 Nov 2021.

Early signs of clinical activity were reported with utomilumab administered at 0.24 mg/kg to 0.6 mg/kg (Merkel cell carcinoma and melanoma), with most responses observed at dose levels ≤1.2 mg/kg (22). Dose levels ≥0.24 mg/kg once every 4 weeks (Q4W) were associated with adequate exposure to utomilumab, and biomarker data (circulating T and NK cells, and soluble 4-1BB/CD137) indicated target modulation at dose levels between 0.24 to 1.2 mg/kg (22). The dose escalation data was consistent with the hypothesis that lower doses of utomilumab may be at least as active as higher doses (22, 25). Here we report the evaluation of two doses (0.24 mg/kg or 1.2 mg/kg) of single-agent utomilumab administered Q4W to patients with metastatic melanoma or metastatic NSCLC. Many of these patients had disease that progressed on treatment with immune checkpoint inhibitors, thereby providing an opportunity to test the hypothesis that 4-1BB/CD137 agonists could convert weak or dysfunctional antitumor immune responses to tumor-controlling immunity. The findings regarding safety, pharmacokinetics, immunogenicity, clinical activity, and biomarker associations with utomilumab treatment are presented here to guide future clinical development of 4-1BB agonists.

## Methods

### Study Design and Treatment

The primary objective of the dose expansion evaluation was to estimate the objective response rate (ORR) to single-agent utomilumab. Secondary objectives were to evaluate safety, pharmacokinetics, immunogenicity, and antitumor effect. Pharmacogenomic evaluation of tumors was an exploratory endpoint.

As the recommended phase II dose had not been established in the dose escalation part of the study, at least 60 patients were to be treated with utomilumab at 0.24 mg/kg or 1.2 mg/kg Q4W. Lower doses could be explored if evidence emerged indicating a greater toxicity than previously observed for the selected dose levels. Utomilumab was administered as a 1-h intravenous infusion Q4W for up to 2 years or until disease progression, unacceptable toxicity, or patient refusal. Premedication could include administration of an antihistamine, an anti-inflammatory agent, or a pain reliever.

### Patients

Adult patients were eligible for enrollment if they had a histological or cytological diagnosis of locally advanced/metastatic melanoma or NSCLC with no available treatment option. Patients with documented disease progression on prior treatment with an immune checkpoint inhibitor (i.e., an anti-CTLA-4 and/or an anti-PD-1/PD-L1 antibody) per Response Evaluation Criteria in Solid Tumors (RECIST) were included in the study. Patients had to provide archival (within 6 months of study start) or baseline tumor biopsies and have Eastern Cooperative Oncology Group performance status ≤1, as well as adequate bone marrow (absolute neutrophil count ≥1.5 x 10^9^/L, platelet count ≥100 x 10^9^/L, hemoglobin >9.0 g/dL), liver (total serum bilirubin ≤1.5 x upper limit of normal [ULN], aspartate aminotransferase and alanine aminotransferase levels ≤2.5 x ULN), renal (serum creatinine ≤2 x ULN or estimated creatinine clearance ≥50 mL/min), and cardiac function.

Patients were not eligible if they had an autoimmune disease (i.e., rheumatoid arthritis, systemic lupus erythematosus, or Crohn’s disease) or other condition impairing the immune system; or a clinically significant bacterial, viral, or fungal infection. Patients were also excluded if they had received prior treatment with an anti-CD137 monoclonal antibody, immunosuppressive therapy with systemic corticosteroids, radiation therapy (within 14 days prior to first dose of study treatment), or other experimental mAbs (within 28 days of study treatment). Furthermore, patients were not eligible if they had known symptomatic brain metastases requiring steroid therapy. However, patients were enrolled in the study if they had completed treatment for brain metastases and recovered from the acute effects of radiation therapy or surgery, had discontinued corticosteroids for ≥4 weeks, and were neurologically stable.

The study was approved by the institutional review board or independent ethics committee of the participating institutions and followed the Declaration of Helsinki and International Conference on Harmonization Good Clinical Practice (ICH GCP) guidelines. The patient informed consent complied with ICH GCP, local regulatory requirements, and legal requirements. The study was sponsored by Pfizer and registered at ClinicalTrials.gov (NCT01307267).

### Study Assessments

#### Safety

Adverse events (AEs) were continuously monitored and characterized by type, frequency, seriousness, timing, and relationship to study drug, and graded by the National Cancer Institute Common Terminology Criteria for Adverse Events v.4.03. Patients were followed for AEs for up to 28 days after the last treatment dose or until all treatment-related toxicities were resolved.

#### Pharmacokinetics and Immunogenicity

Blood samples for pharmacokinetic analysis were collected at multiple, protocol-defined time points: days 1, 7, and 14 of Cycle 1; day 1 of Cycles 2 and 3; days 1, 7, and 14 of Cycle 4; day 1 of every 4 cycles thereafter (for Cycles ≥5); and at the end of treatment. Samples were analyzed using a validated enzyme-linked immunosorbent assay and standard serum pharmacokinetics parameters, including maximum observed serum concentration (*C*_max_), and area under the serum concentration versus time curve (AUC), were estimated for utomilumab using noncompartmental analysis, as previously described (26).

Blood samples for the assessment of antidrug antibodies against utomilumab were collected at protocol-specified time points and analyzed using a validated, electrochemiluminescent bridging assay (26). Antidrug antibody-positive samples were further evaluated for neutralizing antibodies using a cell-based assay (26).

#### Antitumor Activity

Tumor responses were assessed by computed tomography or MRI scans of the chest, abdomen, and pelvis approximately every 8 weeks until Cycle 10, and subsequently every 16 weeks ± 2 weeks, until disease progression, withdrawal from the study, or the end of treatment (if not done in the previous 6 weeks). Partial and complete responses were to be confirmed at least 4 weeks after the initial response, per RECIST v1.1.

#### Biomarker Assessments

Tumor biopsy samples were collected for biomarker assessments from patients before start of therapy and, when considered safe and acceptable to the patient, 4–8 weeks after start of therapy. Chromogenic detection of PD-L1, CD8, 4-1BB, perforin, and granzyme B in formalin-fixed paraffin-embedded sections (Mosaic Laboratories, Lake Forest, CA) was performed as described in Gopal *et al.* (25) using antibodies specific for PD-L1 (E1L3N; Cell Signaling Technology, Danvers, MA), CD8 (C8/144B; Dako, Carpinteria, CA), 4-1BB (BBK-2; Abcam, Cambridge, UK), granzyme B (GrB-7; Dako), and perforin (5B10; Leica, Wetzlar, Germany). Antibodies to perforin and granzyme B were pooled and detected using the same secondary antibody and are therefore labeled as granzyme B + perforin. Assay results were evaluated by image analysis using whole-slide scans (Aperio ImageScope; Leica) except for granzyme B + perforin, which was assessed on manually selected 20x fields.

Analysis of gene expression in tumor biopsies was performed by next-generation sequencing of RNA as previously described (27). Whole-transcriptome profiles were generated using RNAseq (ACE v3; Illumina NovaSeq; San Diego, CA) on formalin-fixed paraffin-embedded tumor tissue. Transcript levels were quantified by the Personalis ACE Cancer Transcriptome Analysis pipeline (Menlo Park, CA), which uses STAR version 2.4.2a-p1 to align reads to the US National Center for Biotechnology Information hs37d5 annotation 105 reference genome and produces transcripts per million (TPM) values for each gene. TPM values were log_2_ transformed for further analysis of individual genes. For volcano plot showing association between expression of protein-coding genes and overall survival, genes with low and/or invariant expression level were filtered out, i.e., genes that were expressed in ≤5% of samples or had standard deviation ≤1 for expression (log_2_ TPM) were removed. RNA sequence data is deposited to Figshare (link).

### *In Vitro* Peripheral Blood Mononulcear Cells (PBMC) Stimulation Assays

*In vitro* PBMC stimulation assays were performed as previously described (21). CD8+ T cells were isolated from the whole blood of healthy donors (EasySep™ Human CD8+ T Cell Isolation Kit, STEMCELL Technologies; Vancouver, Canada) and stimulated with plate bound anti-CD3 (clone UCHT1; BioLegend, San Diego, CA) plus either 0.5 ug/mL cross-linked PF-05082566 or a human IgG2 isotype control antibody. Human IgG2 antibodies were cross-linked with 2.5 to 1 mass to mass addition of goat antihuman IgGFc F(ab′)_2_ (Jackson ImmunoResearch Laboratories, Inc; West Grove, PA). Media samples and total RNA were isolated at 24 h and 48 h. Total RNA samples were isolated using RNAeasy Mini kit (QIAGEN; Germantown, MD) and assayed for differential gene expression using the nCounter GX Human Immunology Kit (NanoString, Seattle, WA). Relative changes in gene expression of PF-05082566 treated samples versus isotype control were determined. A subset of genes corresponding to secreted factors and cell surface receptors demonstrating substantial changes by mRNA expression were further characterized by quantitative reverse transcriptase polymerase chain reaction (Bio-Rad; Hercules, CA) and Luminex xMAP assays (human cytokine 6-plex; MilliporeSigma, Burlington, MA). CD30 upregulation on the cultured CD8+ T cells was measured by flow cytometry using the following antibodies: CD8, RPA-T8, Pacific Blue; CD30, BerH8, PE; CD54, HA58, APC; CD106, 51-10C9, FITC (all from BD Biosciences, San Jose, CA); 4-1BB/CD137, 4B4-1, PerCP-Cy5.5; OX40/CD134, Ber-ACT35, PE-Cy7 (all from BioLegend, San Diego, CA).

### Statistical Analyses

Confirmed ORRs were calculated for all patients and exact two-sided 95% confidence intervals (CIs) were calculated using the Clopper-Pearson method. Time-to-event endpoints (duration of response and progression-free survival [PFS]) were analyzed using the Kaplan–Meier method. Point estimates of Kaplan–Meier rates and median times were presented with their 95% CIs. The CIs for the median were calculated according to the Brookmeyer and Crowley method.

Comparisons of immunohistochemistry biomarkers (PD-L1, CD8, 4-1BB, granzyme B + perforin) between disease control (best overall response [BOR] of complete response, partial response, or stable disease) and disease progression (BOR of progressive disease) subgroups were performed using the Wilcoxon rank sum test. Associations between gene expression and disease control were determined using odds ratio of disease control for one unit increase in biomarker. Differences in gene expression between paired pretreatment and on-treatment biopsies are reported as log_2_(fold change).

## Results

### Patients and Treatment

Sixty-three patients, 43 with metastatic melanoma and 20 with advanced NSCLC, who had mostly progressed on prior immune checkpoint inhibitor treatment per RECIST, received intravenous utomilumab Q4W at 0.24 (*n* = 36), 1.2 (*n* = 26), and 10 mg/kg (*n* = 1). Patient demographic and baseline characteristics are summarized in **Supplementary Tables S1** and **S2**.

Overall, 63.5% of patients were men, with a median age of 65 years (range, 24–79 years). Thirty-four (54.0%) patients were ≥65 years of age. Thirty-five (55.6%) patients had received prior therapy for advanced/metastatic disease, including 20 (31.7%) patients with three or more prior lines of treatment **Supplementary Table S3**. In the melanoma cohort 15 patients had received both an anti-CTLA4 and an anti-PD-1/PD-L1, 16 patients had received an anti-PD-1/PD-L1, and 5 patients had received an anti-CTLA4. In the NSCLC cohort 14 patients had received an anti-PD-1/PD-L1. The most common prior immune checkpoint inhibitor therapy for NSCLC was nivolumab (in 70% of patients) and pembrolizumab for melanoma (60.5%), followed by ipilimumab plus nivolumab (30.2%). One patient each (1.6%) with NSCLC was previously treated with atezolizumab or durvalumab. The median duration since the last immune checkpoint inhibitor dose, prior to study entry, was 239 days (range 70-959 days).

### Safety

Overall, 55 (87.3%) patients developed a treatment-emergent AE and 32 (50.8%) patients developed a treatment-related AE of any grade. Treatment with single-agent utomilumab was generally well tolerated at all dose levels as most of the treatment-related AEs reported were grade 1 or 2 (**Table 1**). The most common treatment-related AEs, observed in >5% of patients, were fatigue (15.9%), nausea (15.9%), diarrhea (9.5%), headache (6.3%) and elevated aspartate aminotransferase (6.3%).

**Table 1:** Treatment-related adverse events reported in ≥4% of patients.

AE^a^	Grade 1	Grade 2	Grade 3	Grade 4	Total
Any AE	17 (27.0)	12 (19.0)	2 (3.2)	1 (1.6)	32 (50.8)
Fatigue	6 (9.5)	4 (6.3)	0	0	10 (15.9)
Nausea	6 (9.5)	4 (6.3)	0	0	10 (15.9)
Diarrhea	3 (4.8)	2 (3.2)	1 (1.6)	0	6 (9.5)
Headache	3 (4.8)	1 (1.6)	0	0	4 (6.3)
Pyrexia	2 (3.2)	1 (1.6)	0	0	3 (4.8)
Decreased appetite	2 (3.2)	1 (1.6)	0	0	3 (4.8)
Vomiting	2 (3.2)	1 (1.6)	0	0	3 (4.8)
Rash	3 (4.8)	0	0	0	3 (4.8)
Maculopapular rash	2 (3.2)	1 (1.6)	0	0	3 (4.8)

a Two patients had grade 3 AEs which included diarrhea (one instance), hyponatremia (one instance) and colitis (one instance), and one patient had grade 4 hyperbilirubinemia. No patient experienced a grade 5 treatment-related AE.

Two (3.2%) patients experienced grade 3 treatment-related AEs (i.e., diarrhea, colitis, and hyponatremia). One patient each (1.6%) had a treatment-related AE of grade 1 and grade 4 hyperbilirubinemia. The latter AE occurred in Cycle 2 in a 67-year-old patient with NSCLC (0.24 mg/kg dose group) and hepatic metastases. The patient had received adjuvant therapy with pemetrexed, cisplatin, and nivolumab in the year prior to study entry, and docetaxel plus the VEGF receptor inhibitor ramucirumab until ~3 months before start of study treatment, which may have affected hepatic function (28, 29). There were no treatment-related deaths. One patient with NSCLC died of a treatment-emergent cardio-respiratory arrest deemed related to the underlying disease. Overall, 18 (28.6%) patients had a serious treatment-emergent AE. Treatment with utomilumab was permanently discontinued in five (7.9%) patients due to treatment-emergent AEs (cardio-respiratory arrest, colitis, enterocolitis, hyperbilirubinemia).

### Pharmacokinetics and Immunogenicity

Utomilumab pharmacokinetic parameters for single-dose administration at 0.24 mg/kg and 1.2 mg/kg are summarized in **Supplementary Table S4**. Utomilumab serum exposure (*C*_max_ and AUC_inf_) increased with increasing doses.

Eight patients had positive antidrug antibodies against utomilumab at baseline, likely due to pre-existing host antibodies cross-reacting with utomilumab. Thirty-one (46.3%) patients exhibited treatment-induced antidrug antibodies, and none were treatment-boosted. In addition, 13 (19.4%) patients exhibited treatment-induced neutralizing antibodies.

### Clinical Activity

**Figure 1:**
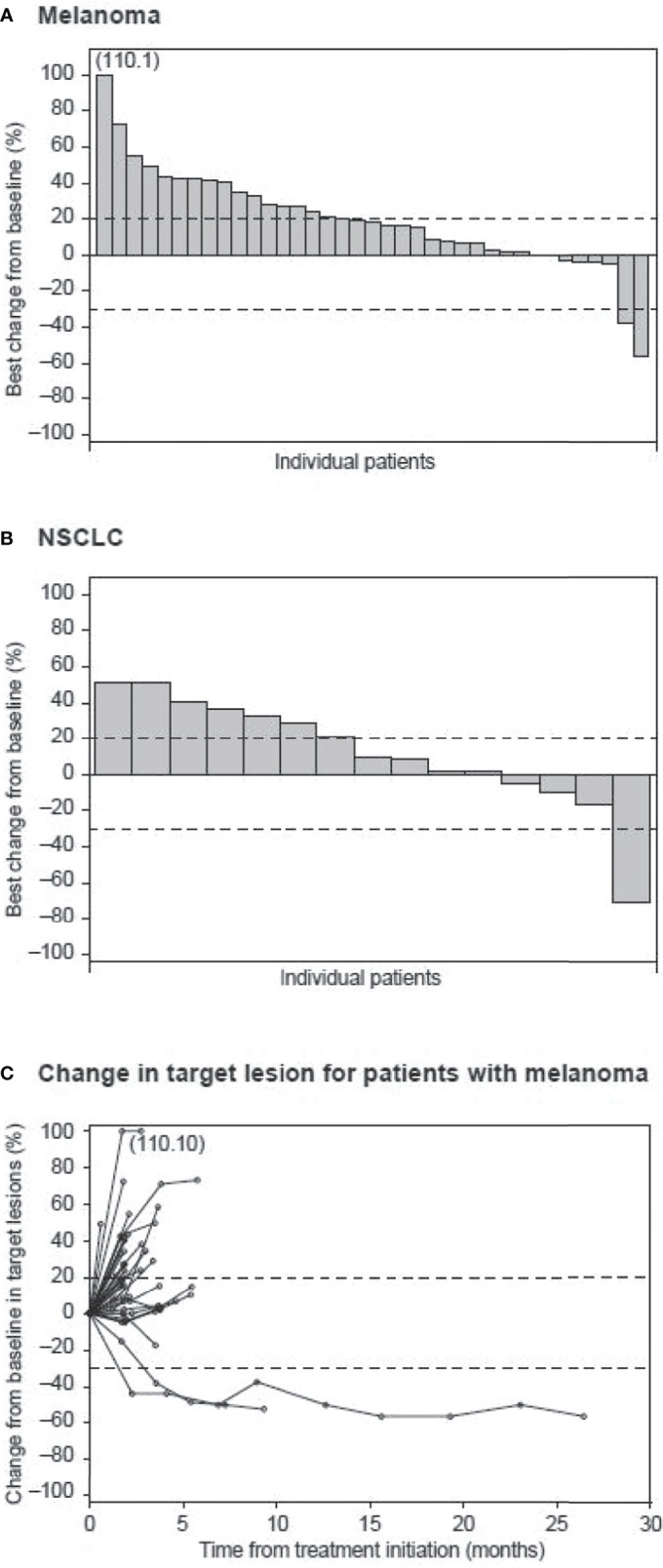
Waterfall plot of best percent change from baseline in patients with **(A)** melanoma and **(B)** NSCLC. Dashed lines indicate a 30% decrease and a 20% increase from baseline in the sum of the longest diameters of target lesions. **(C)** Spider plot of percent change from baseline in target lesions of patients with melanoma over time.

Of the 43 patients with metastatic melanoma who received utomilumab, one (2.3%) patient (0.24 mg/kg dose group) had a BOR of confirmed partial response that lasted for more than 24 months (**Figures 1A and C**). This patient had completed prior treatment with pembrolizumab (4 cycles) until ~2 months before commencing study treatment, with BOR of disease progression. A second patient in this utomilumab 0.24 mg/kg dose group, whose disease had previously progressed after multiple therapies including ipilimumab, nivolumab, and pembrolizumab, had a confirmed partial response in target lesions at Cycles 4 through 10 but subsequently developed progressive disease in nontarget lesions. Ten (23.3%) patients with melanoma had a BOR of stable disease. The confirmed ORR in the melanoma cohort was 2.3% (95% exact CI: 0.1–12.3) (**Table 2**).

**Table 2:** Best overall response by tumor type.

*n* (%)	Melanoma *n* = 43	NSCLC *n* = 20	Total *N* = 63
CR	0	0	0
PR	1 (2.3)	0	1 (1.6)
SD	10 (23.3)	10 (50.0)	20 (31.7)
Objective progression	31 (72.1)	6 (30.0)	37 (58.7)
Not evaluable	1 (2.3)	4 (20.0)	5 (7.9)
ORR (CR + PR) (95% exact CI)	1 (2.3) (0.1–12.3)	0 (0.0–16.8)	1 (1.6) (0.0–8.5)

CR, complete response; PR, partial response; SD, stable disease.

Of the 20 patients with advanced NSCLC, one patient (0.24 mg/kg dose group) had a partial response after 4 months of study treatment that was not confirmed (**Figure 1B**). This patient had received prior treatment with cisplatin/etoposide and nivolumab (one cycle each), with nivolumab administration ending ~1 month before study entry, with BOR of progressive disease. No confirmed partial or complete response was observed in the NSCLC cohort. However, 10 (50%) patients had a BOR of stable disease.

Median PFS was 1.8 (95% CI: 1.7–1.9) months in patients with melanoma and 3.6 (95% CI: 1.6–6.5) months in those with NSCLC.

### Biomarkers, Clinical Outcomes, and Utomilumab Dose

Due to the relatively small cohort size and low ORR, the expansion cohorts had limited statistical power to identify biomarkers associated with utomilumab treatment (30). For the purpose of exploratory hypothesis generation, two categorical subgroup analyses were performed: (*a*) disease control (defined as a BOR of complete or partial response or stable disease) versus disease progression (defined as a BOR of disease progression) in melanoma and in NSCLC patients, and (*b*) utomilumab 0.24 mg/kg versus 1.2 mg/kg in patients with melanoma.

**Figure 2:**
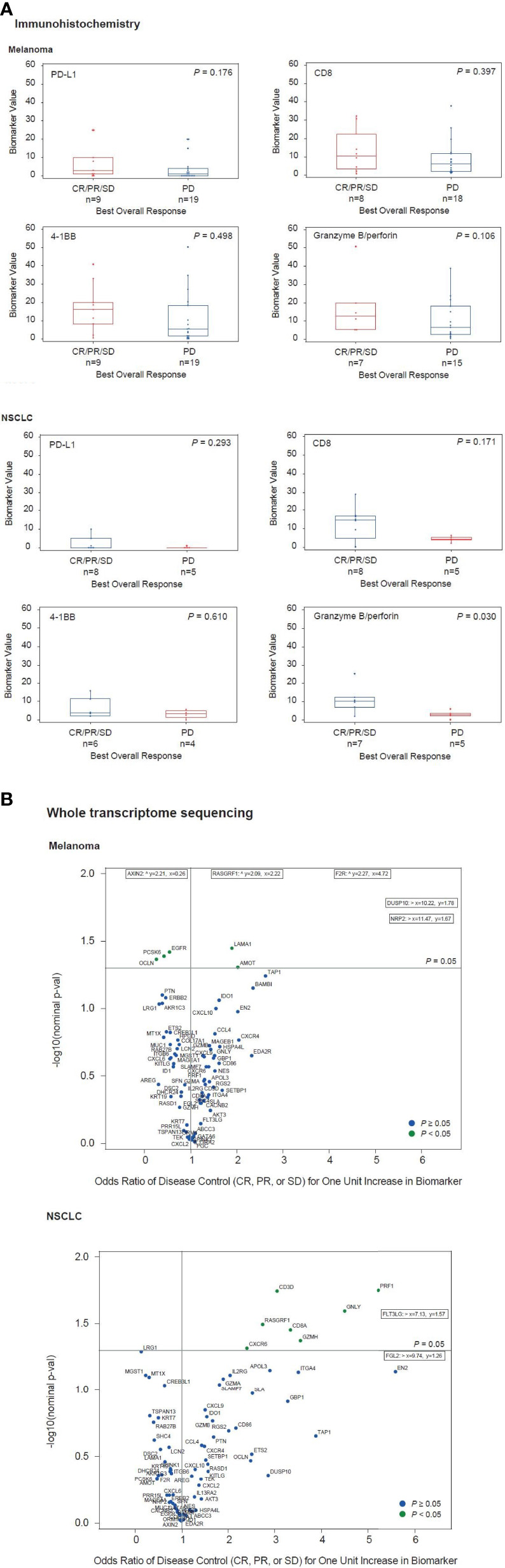
Associations between disease control vs disease progression and tumor biomarkers measured by **(A)** immunohistochemistry and **(B)** whole transcriptome sequencing.

The percentage of cells staining with PD-L1, CD8, 4-1BB, and granzyme B plus perforin in patients with disease control and disease progression from melanoma (n=28) and NSCLC cohorts (n=13) are shown in **Figure 2A** and **Supplementary Table S5**. Average values for each biomarker were higher in the disease control versus disease progression subgroups of patients for both melanoma and NSCLC cohorts, but *P* values were >0.05 in all comparisons except for granzyme B plus perforin in NSCLC (*P* = 0.03). Use of RNA-seq data from tumor biopsies collected prior to or on the first day of utomilumab treatment (baseline) enabled the identification of genes that were differentially expressed in the disease control and disease progression subgroups (**Figure 2B** and **Supplementary Table S6**). Genes that were positively associated with disease control (n=3) in the NSCLC subgroup (total n=9) represented multiple immune-related functions, including some that have been associated with improved outcome from immune checkpoint inhibitor therapy, such as *CD8A*, *GZMB*, *PRF1*, (consistent with the immunohistochemistry results), *CXCL9*, *CXCL10*, *CXCR6*, *GZMA*, and *IDO1* (**Figure 2B**) (27, 31–33). Similar trends, albeit smaller, were observable for the subgroup of patients with disease control (n=6) in melanoma (total n=22) (**Figure 2B**). Taken together, these results are consistent with the hypothesis that clinical benefit from utomilumab may be contingent in part on a pre-existing “inflamed” phenotype (34).

**Figure 3:**
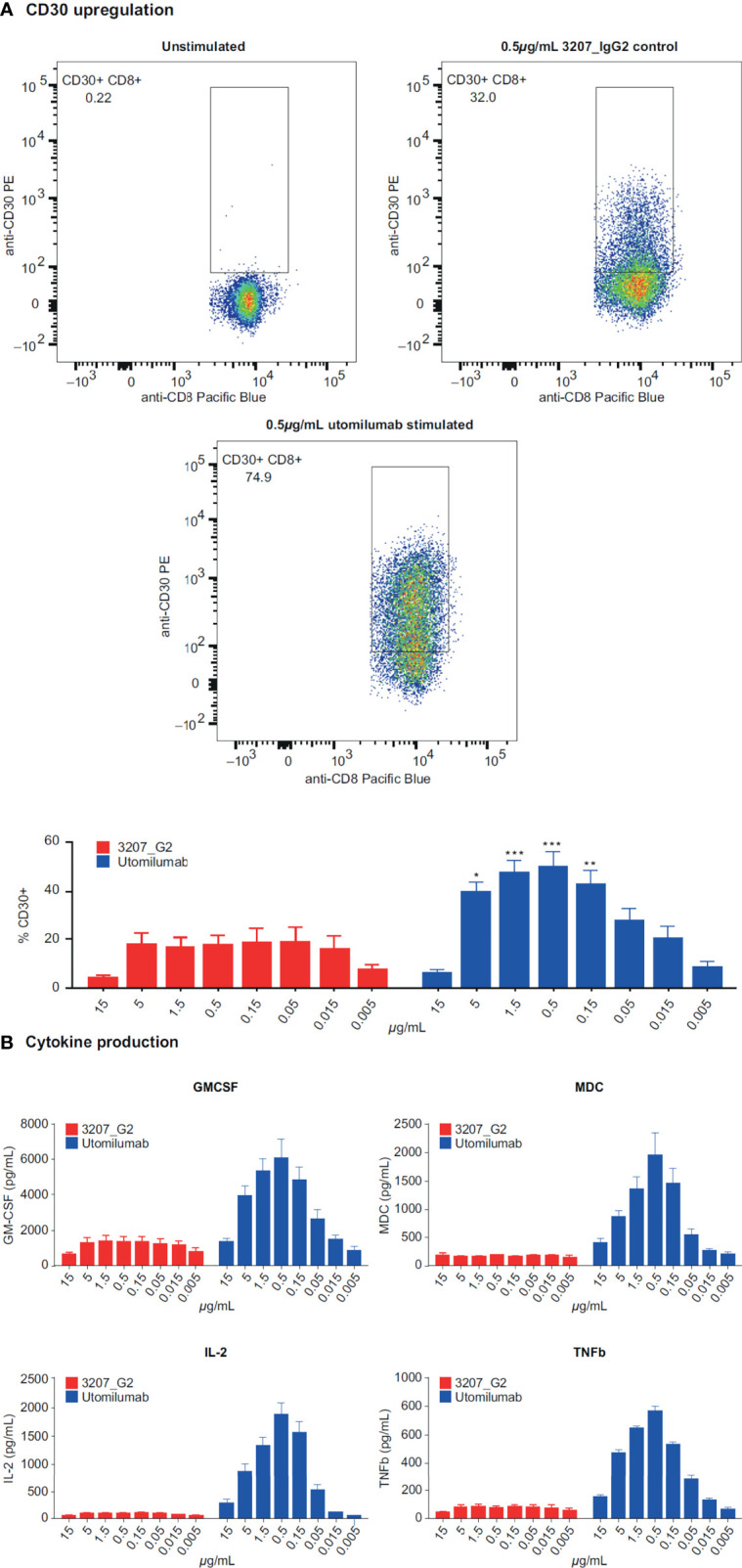
Response of CD3-activated human CD8+ T cells to varying concentrations of utomilumab measured by **(A)** CD30 upregulation on the cell surface and **(B)** cytokine production. GMCSF, granulocyte-macrophage colony-stimulating factor; IL-2, interleukin-2; MDC, macrophage-derived chemokine; TNFb, tumor necrosis factor beta *p < 0.05; **p < 0.01; ***p < 0.005 vs isotype control at the same concentration.

**Figure 4:**
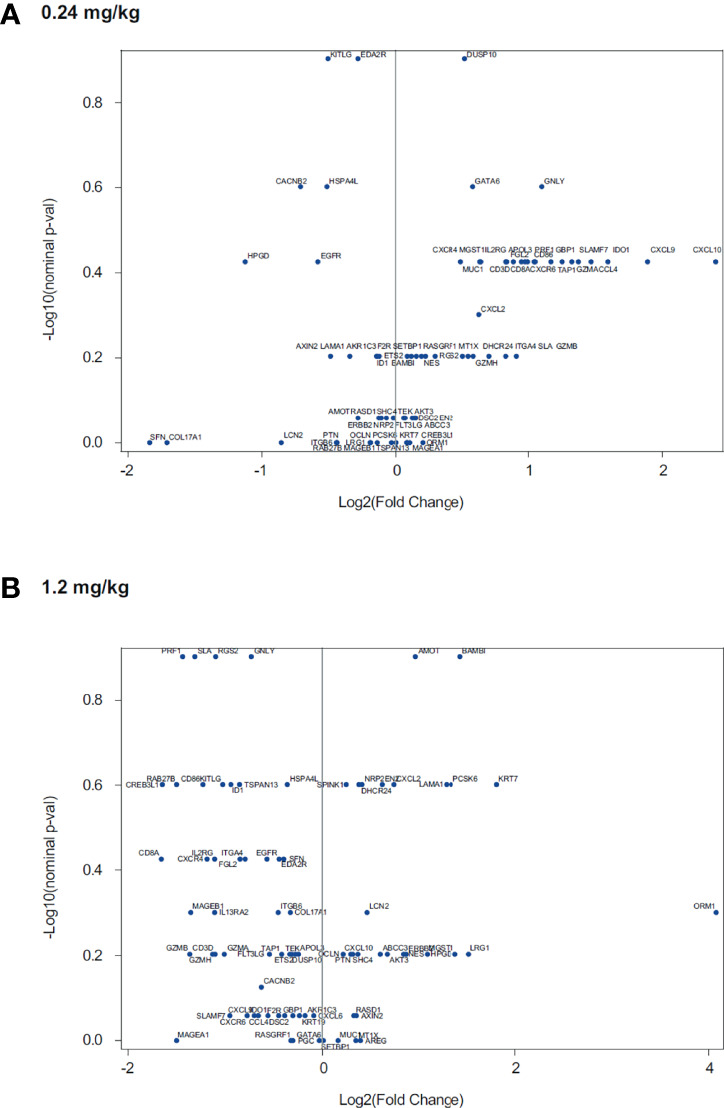
Association of utomilumab dose with fold-change in gene expression in paired tumor biopsies for treatment with utomilumab **(A)** 0.24 mg/kg and **(B)** 1.2 mg/kg.

Evaluation of data from *in vitro* stimulation of CD8+ T cells with anti-CD3 plus or minus different doses of utomilumab compared with isotype control suggested that lower concentrations of utomilumab were more effective than higher ones (**Figure 3**; **Supplementary Figure S1**). Differential gene expression analysis of limited numbers of paired baseline and on-treatment biopsies collected from melanoma patients treated with utomilumab 0.24 mg/kg (total n = 4, 2 patients displaying disease control) and 1.2 mg/kg (total n = 4, no patients displaying disease control) is shown in **Figure 4**. Modest increases in the expression of the immune response–related genes noted above, as well as other immune-related genes such as *CCL4*, *SLAMF7*, *TAP1*, and *GBP1*, were observed at 0.24 mg/kg (**Figure 4A** and **Supplementary Table S6**) but not at 1.2 mg/kg doses (**Figure 4B** and **Supplementary Table S6**). These results support further evaluation of the hypothesis that lower doses of 4-1BB agonist mAbs may be more pharmacologically active than higher doses.

## Discussion

In this dose-expansion cohort study, we compared 0.24 mg/kg and 1.2 mg/kg dose levels of the 4-1BB/CD137 agonist antibody utomilumab with respect to safety, antitumor activity, pharmacokinetics, and biomarkers in patients with metastatic melanoma and NSCLC. The two dose levels showed pharmacokinetic properties expected from the dose escalation cohorts, and they displayed comparable safety and tolerability in both melanoma and NSCLC tumor types (22, 25). Immunogenicity analyses showed the incidence of baseline and treatment-induced antidrug antibodies against utomilumab in the expansion cohorts was comparable to that observed in patients treated with utomilumab in the dose escalation part of the trial (22). Comparable safety (including incidence of hypersensitivity/infusion reactions) and pharmacokinetic profiles were previously reported for antidrug antibody/neutralizing antibody-negative and -positive patients with solid tumors following administration of utomilumab (22). Neither dose level of single-agent utomilumab demonstrated a notable ORR.

A few confirmed and unconfirmed partial responses by RECIST1.1 were observed in patients with disease progression after prior treatment with a PD-1–targeted immune checkpoint inhibitor (i.e., pembrolizumab and nivolumab) as well as other anticancer agents. One patient with melanoma treated with 0.24 mg/kg utomilumab maintained a partial response for ~2 years. In addition, 23% of patients with melanoma and 50% of those with NSCLC had a best response of stable disease. These results are consistent with prior evaluation of utomilumab in solid tumors (22, 26, 35). Although treatment-unrelated objective responses or disease stabilization are not expected in these disease settings, the study design does not enable conclusive demonstration that the clinical benefit is due to utomilumab.

**Figure 5:**
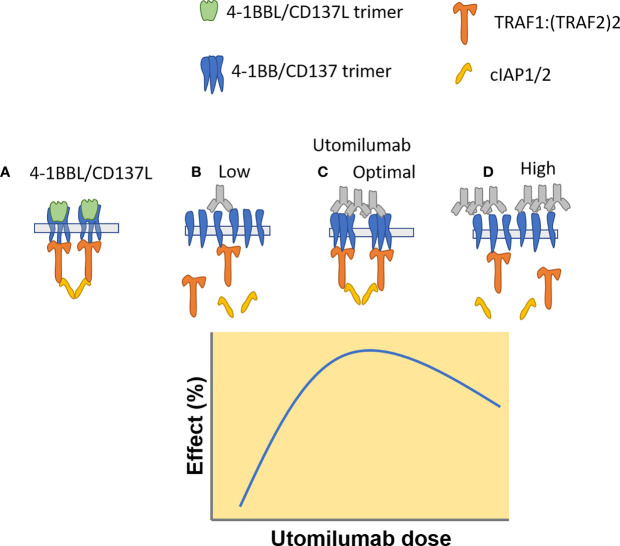
Proposed mechanism of utomilumab – mediated 4-1BB/CD137 signaling, based on a model proposed by Zapata et al. (https://doi.org/10.3389/fimmu.2018.02618). **(A)** CD137L trimers induce formation of CD137 trimers and TRAF – cIAP1/2 signaling complexes. **(B)** Low concentrations of utomilumab fail to enable formation of the 4-1BB/CD137 signaling complex. **(C)** Optimum concentrations of utomilumab enable complex formation. **(D)** High concentrations of utomilumab disrupt complex formation.

An extensive body of literature describes the positive association between response to immune checkpoint inhibitors and immune system biomarkers in the tumor, including PD-L1, the interferon-g pathway, and cytotoxic cells (36–38). Early preclinical tumor models suggested that agonists to 4-1BB/CD137 could amplify antitumor immune responses and thereby elicit clinical benefit for patients whose immune responses were suboptimal (4, 5, 16). In spite of the low statistical power inherent in small cohorts of patient, we did see some evidence that clinical benefit from single-agent utomilumab was positively associated with biomarkers of T cells, cytotoxicity, and IFNg responses in NSCLC tumors. Transcriptional profiling of paired pretreatment and on-treatment biopsies uncovered increased expression of genes related to antitumor activity in patients treated with utomilumab at 0.24 mg/kg relative to 1.2 mg/kg. The greater pharmacodynamic activity at the lower dose is consistent with *in vitro* analysis of human CD8+ T cells, which also suggested that higher doses of utomilumab may be suboptimal. The dose-response relationship for 4-1BB agonist mAbs has been unclear. Signaling through 4-1BB has been proposed to be contingent on formation of a structured signalosome (39), and it is possible that formation of such signalosomes may be impeded at higher doses of utomilumab (**Figure 5**).

Although utomilumab showed some signs of the expected agonist mechanisms in patients, the low response rates suggest that either the signaling or the clinical setting was suboptimal. The relationship between 4-1BB/CD137 engagement *in situ* and downstream signaling is complex (4, 39–41) and it is plausible that utomilumab engagement of 4-1BB/CD137, even at the optimum concentration, may not assemble a signaling complex with sufficient activity (23). Most patients in this study had progressed on prior checkpoint-containing therapy, and tumors in such patients may have one or more resistance mechanisms to antitumor immunity (42, 43) that cannot be overcome by 4-1BB/CD137 agonists. 4-1BB/CD137 remains a potential target for cancer immunotherapy; however, careful attention should be given to optimizing dose, signaling mechanism, and clinical setting in future clinical studies.

## Data Availability Statement

The original contributions presented in the study are publicly available. This data can be found here: https://www.ncbi.nlm.nih.gov/geo/, accession number GSE208858.

## Ethics Statement

The studies involving human participants were reviewed and approved by local IRBs. The patients/participants provided their written informed consent to participate in this study.

## Author Contributions

Conceptualization: All authors; Data acquisition: DH, AG, AS, SP, AH, TD, SR, AP, SS, TF, KF, and AR; Data analysis and interpretation: BH, KF, YC, CD, and AR; Supervision of data analysis: YC, BH, KF, CD, and AR; Manuscript drafting: all authors. Manuscript review and approval for submission: all authors.

## Funding

Ying Chen, Craig B. Davis, Timothy S. Fisher, Bo Huang, and Kolette D. Fly are employed by Pfizer. This study received funding from Pfizer. The funder was involved in the analysis and interpretation of data, decision to publish, and in the preparation of the manuscript. DSH acknowledges CCSG and CTSA grants to MD Anderson: P30 CA016672 and UL1 TR003167. ANS acknowledges the NCI Core Center Grant for Memorial Sloan Kettering: P30 CA008748.

## Conflict of Interest

DH has disclosed consulting/advisory roles for Alpha Insights, Axiom, Adaptimmune, Baxter, Bayer, Genentech, GLG, Group H, Guidepoint Global, Infinity, Janssen, Merrimack, Medscape, Numab, Pfizer, Seattle Genetics, Takeda, and Trieza Therapeutics; research funding from AbbVie, Adaptimmune, Amgen, Astra Zeneca, Bayer, Bristol Myers Squibb, Daiichi Sankyo, Eisai, Fate Therapeutics, Genentech, Genmab, Ignyta, Infinity, Kite, Kyowa, Lilly, Loxo, Merck, MedImmune, Mirati, MiRNA, Molecular Templates, Mologen, National Cancer Institute/Cancer Therapy Evaluation Program, Novartis, Pfizer, Seattle Genetics, and Takeda; travel/accomodations expense reimbursement from Loxo and MiRNA; and other ownership interests in Molecular Match (advisor), OncoResponse (founder), and Presagia Inc. (advisor). AG received research funding from Bristol Myers Squibb, Gilead, Janssen, Merck, Pfizer, Seattle Genetics, and Takeda; and consulting honoraria from Amgen, Acerta, Bayer, Brim Biotechnologies, Gilead, Janssen, and Seattle Genetics. AS received consulting honoraria from Bristol Myers Squibb, Novartis, and Immunocore; and research funding from Bristol Myers Squibb, Immunocore, Novartis, Xcovery, Targovax ASA, Polaris, Pfizer, Checkmate Pharmaceuticals, and Foghorn Therapeutics. SP received research funding from Bristol Myers Squibb, Eli Lilly, Incyte, MedImmune, Pfizer, Roche/Genentech, and Xcovery; and speaker honoraria from Boehringer Ingelheim and Merck. AH served as consultant/advisor for AstraZeneca, Bayer, Bristol Myers Squibb, Eisai, Genentech, and Merck; received honoraria from Bayer, Bristol Myers Squibb, Eisai, and Exelixis; and research funding from Genentech and Merck Serono. TD served as consultant/advisor for AbbVie, Amgen, Bayer, Boehringer Ingelheim, Daiichi Sankyo, MSD, Novartis, Rakuten Medical, Sumitomo Dainippon, Taiho, and Takeda; received honoraria from AbbVie, Astellas, Bristol Myers Squibb, Oncolys Biopharma, Ono, and Taiho; and institutional research funding from Abbvie, Bristol Myers Squibb, Boehringer Ingelheim, Daiichi Sankyo, Eisai, Kyowa Hakko Kirin, Lilly, Merck Serono, MSD, Novartis, Pfizer, Quintiles, Sumitomo Dainippon, and Taiho. SR served as consultant/advisor for Amgen, Astra Zeneca, Bristol Myers Squibb, Merck, Roche, Takeda, and Tesaro; and received institutional research funding from Advaxis, Amgen, Astra Zeneca, Bristol Myers Squibb, Genmab, Merck, Takeda, and Tesaro. AP has received institutional research funding from Arcus, Exelixis, Surface Oncology, Upsher-Smith, Lilly, Daiichi Sankyo, Merck, Tesaro, Forty Seven, Bolt, Pieris Pharmaceuticals, Vigeo Therapeutics, Syndax, Seattle Genetics, Pfizer, Livzon, Klus Pharma, Fochon, Gilead Sciences, Plexxikon, Corvus Pharmaceuticals, Five Prime Therapeutics, Infinity Pharmaceuticals, Ionova, Abbvie, Astellas Pharma, Symphogen, Sanofi, honoraria from the Texas Society of Clinical Oncology and served as a consultant/advisor for Novartis, Gilead, Seattle Genetics, Silverback Therapeutics, Bayer, Daiichi Sankyo, Inc, Shenzen IONOVA Life Sciences Co., Ltd., and Merck. SS received research funding from Advanced Accelerator Applications (Novartis), Amgen, Astra Zeneca, Genentech and MSD; and honoraria to the institution from Astra Zeneca, Bristol Myers Squibb, MSD, and Novartis. YC, CD, BH, KF, and A. D. Thall are employees of and own stock in Pfizer Inc. AR disclosed consulting honoraria from Amgen, Bristol Myers Squibb, Chugai, Genentech, Merck, Novartis, Roche, and Sanofi; membership in scientific advisory boards and stocks in Advaxis, Arcus Biosciences, Bioncotech Therapeutics, Compugen, CytomX, Five Prime, FLX-Bio, ImaginAb, Isoplexis, Kite/Gilead, Lutris Pharma, Merus, PACT Pharma, Rgenix and Tango Therapeutics; and research funding from Agilent and Bristol Myers Squibb.

The remaining authors declare that the research was conducted in the absence of any commercial or financial relationships that could be construed as a potential conflict of interest.

## Publisher’s Note

All claims expressed in this article are solely those of the authors and do not necessarily represent those of their affiliated organizations, or those of the publisher, the editors and the reviewers. Any product that may be evaluated in this article, or claim that may be made by its manufacturer, is not guaranteed or endorsed by the publisher.
