# Supplementary material for: Utomilumab in Patients With Immune Checkpoint Inhibitor-Refractory Melanoma and Non-Small-Cell Lung Cancer
Source: Front Immunol. 2022 Aug 2;13:897991. doi: 10.3389/fimmu.2022.897991 (PMC9379324; doi:10.3389/fimmu.2022.897991)
Supplement: Supplementary file 1 [file DataSheet_1.pdf]

**Supplementary Figure S1 Response of CD3-activated human CD8+ T cells to varying concentrations of utomilumab measured by (A) targeted gene expression panel and (B) quantitative RT-PCR**

**A**

**Nanostring Summary Data**
**Fold from isotype (>30% increase)**

| 24h Nanostring | | 48h Nanostring | |
|---|---|---|---|
| ***IL2*** | **4.17** | ***IL9*** | **4.48** |
| ***CCL22*** | **2.63** | ***CCL22*** | **4.47** |
| ***CSF2*** | **2.19** | ***IL2*** | **3.54** |
| *IL23A* | 1.88 | ***IL3*** | **2.06** |
| *TNFRSF8* | 1.86 | ***TNFRSF8*** | **2.01** |
| *IL3* | 1.76 | *IL13* | 1.90 |
| *LTA* | 1.63 | *CSF2* | 1.86 |
| *IL13* | 1.50 | *CCL20* | 1.83 |
| *LIF* | 1.49 | *LIF* | 1.83 |
| *ICAM1* | 1.49 | *IL1A* | 1.69 |
| *NFKBIA* | 1.49 | *LTA* | 1.61 |
| *IL9* | 1.46 | *EB13* | 1.56 |
| *CD83* | 1.41 | *PLAUR* | 1.53 |
| *TRAF1* | 1.40 | *BATF3* | 1.51 |
| *NFKB2* | 1.40 | *IL1R2* | 1.51 |
| *TNFRSF4* | 1.38 | *IL23A* | 1.49 |
| *CCL20* | 1.37 | *FADD* | 1.49 |
| *VCAM1* | 1.31 | *SOCS3* | 1.46 |
| | | *ICAM1* | 1.38 |
| | | *CSF1* | 1.37 |
| | | *TRAF1* | 1.34 |
| | | *HLA-DOB* | 1.33 |
| | | *VCAM1* | 1.33 |
| | | *CDKN1A* | 1.32 |
| | | *IL10* | 1.32 |
| | | *BCL3* | 1.31 |
| | | *IL21* | 1.31 |
| | | *NFKBIA* | 1.31 |
| | | *NFKB2* | 1.30 |

**B**

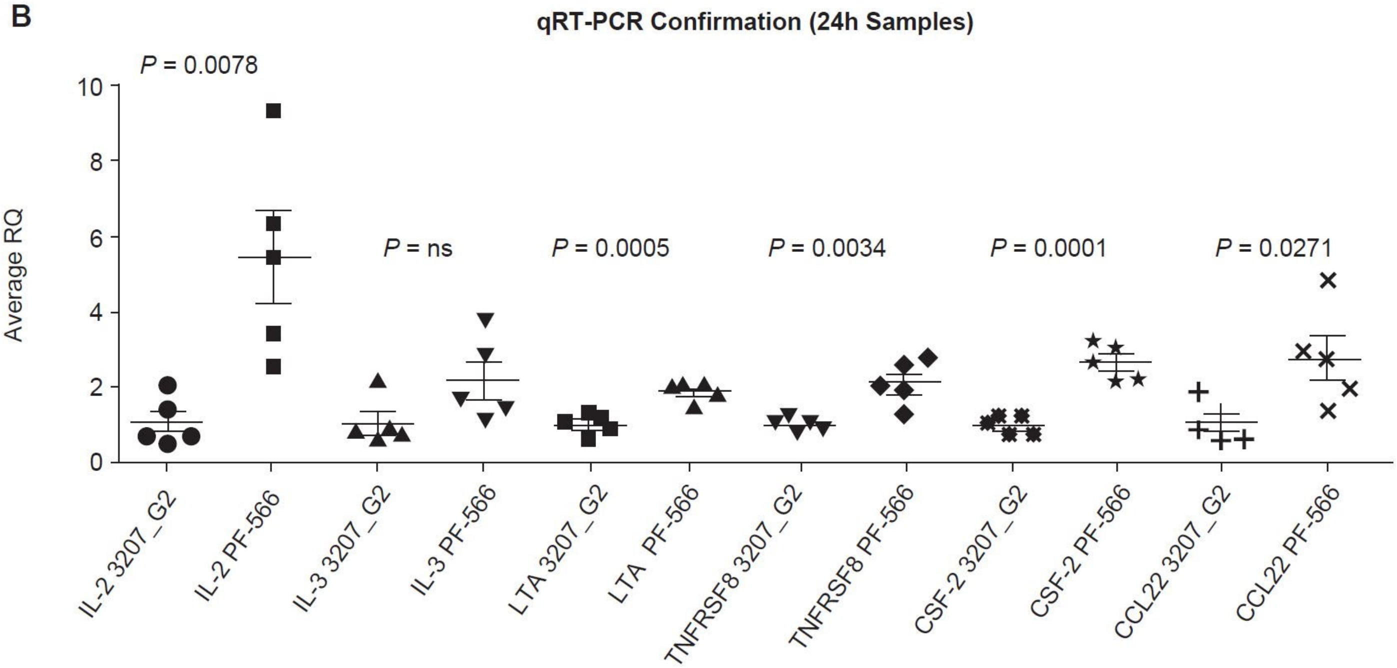


ns=not significant; qRT-PCR, quantitative reverse transcription polymerase chain reaction
