# Supplementary material for: Utomilumab in Patients With Immune Checkpoint Inhibitor-Refractory Melanoma and Non-Small-Cell Lung Cancer
Source: Front Immunol. 2022 Aug 2;13:897991. doi: 10.3389/fimmu.2022.897991 (PMC9379324; doi:10.3389/fimmu.2022.897991)
Supplement: Supplementary file 2 [file Table_1.pdf]

SUPPLEMENTARY INFORMATION

Supplementary Table S1. Patient demographics and baseline characteristics

	Utomilumab		
	**Melanoma** ***n*** **= 43**	**NSCLC** ***n*** **= 20**	**Total** ***N*** **= 63**
Male : Female, *n* (%)	29 (67.4) : 14 (32.6)	11 (55.0) : 9 (45.0)	40 (63.5) : 23 (36.5)
Median age (range), years	59.0 (24–79)	67.5 (45–75)	65.0 (24–79)
≥65 years, *n* (%)	18 (41.9)	16 (80.0)	34 (54.0)
Race, *n* (%)			
White	35 (81.4)	17 (85.0)	52 (82.5)
Black	0	2 (10.0)	2 (3.2)
Asian	1 (2.3)	1 (5.0)	2 (3.2)
Other	7 (16.3)	0	7 (11.1)
Prior therapies for advanced/ metastatic disease, *n* (%)			
0	22 (51.2)	6 (30.0)	28 (44.4)
1	4 (9.3)	3 (15.0)	7 (11.1)
2	8 (18.6)	0	8 (12.7)
3	1 (2.3)	3 (15.0)	4 (6.3)
>3	8 (18.6)	8 (40.0)	16 (25.4)
Most recent ICI prior to study treatment, *n* (%)			
Atezolizumab	0	1 (5.0)	1 (1.6)
Durvalumab	0	1 (5.0)	1 (1.6)
Ipilimumab	13 (30.2)	0	13 (20.6)
Nivolumab	13 (30.2)	14 (70.0)	27 (42.9)
Pembrolizumab	26 (60.5)	3 (15.0)	29 (46.0)
Median duration since last ICI dose at study entry (range), days	225 (70–959)	276 (91–694)	239 (70–959)

Abbreviation: ICI, immune checkpoint inhibitors CTLA-4 or PD-1/PD-L1; NSCLC, non-small-cell lung cancer; PD-1, programmed cell death receptor 1; PD-L1, programmed cell death receptor ligand 1.
