# Supplementary material for: Utomilumab in Patients With Immune Checkpoint Inhibitor-Refractory Melanoma and Non-Small-Cell Lung Cancer
Source: Front Immunol. 2022 Aug 2;13:897991. doi: 10.3389/fimmu.2022.897991 (PMC9379324; doi:10.3389/fimmu.2022.897991)
Supplement: Supplementary file 5 [file Table_4.pdf]

Supplementary Table S4. Pharmacokinetic parameters for single-agent utomilumab (cycle 1)[a]

Dose (mg/kg)	*N*, *n* [b]	C_{max} (μg/mL)	AUC_{inf} (μg·hr/mL)	CL (mL/hr/kg)	V_{ss} (mL/kg)	$t_{1/2}$ (day)
0.24	36, 12	3.17 (30)	651 (42)	0.369 (42)	118 (23)	11.2 (5.33)
1.2	26, 18	17.4 (23)	2881 (29)	0.417 (29)	122 (21)	9.21 (2.11)

[a] Geometric mean (geometric % CV) for C_{max}, AUC_{inf}, CL, and V_{ss}. Arithmetic mean (± SD) for $t_{1/2}$.

[b] *N* denotes the number of patients contributing to the summary statistics for C_{max} while *n* denotes number of patients contributing to the summary statistics for AUC_{inf}, CL, V_{ss}, and $t_{1/2}$.

AUC_{inf}, area under the serum concentration-time curve from time 0 to infinity; CL, clearance; C_{max}, maximum observed serum concentration; CV, coefficient of variation; SD, standard deviation; $t_{1/2}$, terminal half-life; V_{ss}, volume of distribution at steady state.
